# Supplementary material for: Unraveling the molecular architecture of autoimmune thyroid diseases at spatial resolution
Source: Nat Commun. 2024 Jul 13;15:5895. doi: 10.1038/s41467-024-50192-5 (PMC11246508; doi:10.1038/s41467-024-50192-5)
Supplement: Supplementary file 1 — Supplementary Information [file 41467_2024_50192_MOESM1_ESM.pdf]

Unraveling the molecular architecture of autoimmune thyroid diseases at spatial resolution

Rebeca Martínez-Hernández, Nuria Sánchez de la Blanca, Pablo Sacristán-Gómez, Ana Serrano-Somavilla, José Luis Muñoz De Nova, Fátima Sánchez Cabo, Holger Heyn, Miguel Sampedro-Núñez and Mónica Marazuela

SUPPLEMENTARY FIGURES AND TABLES

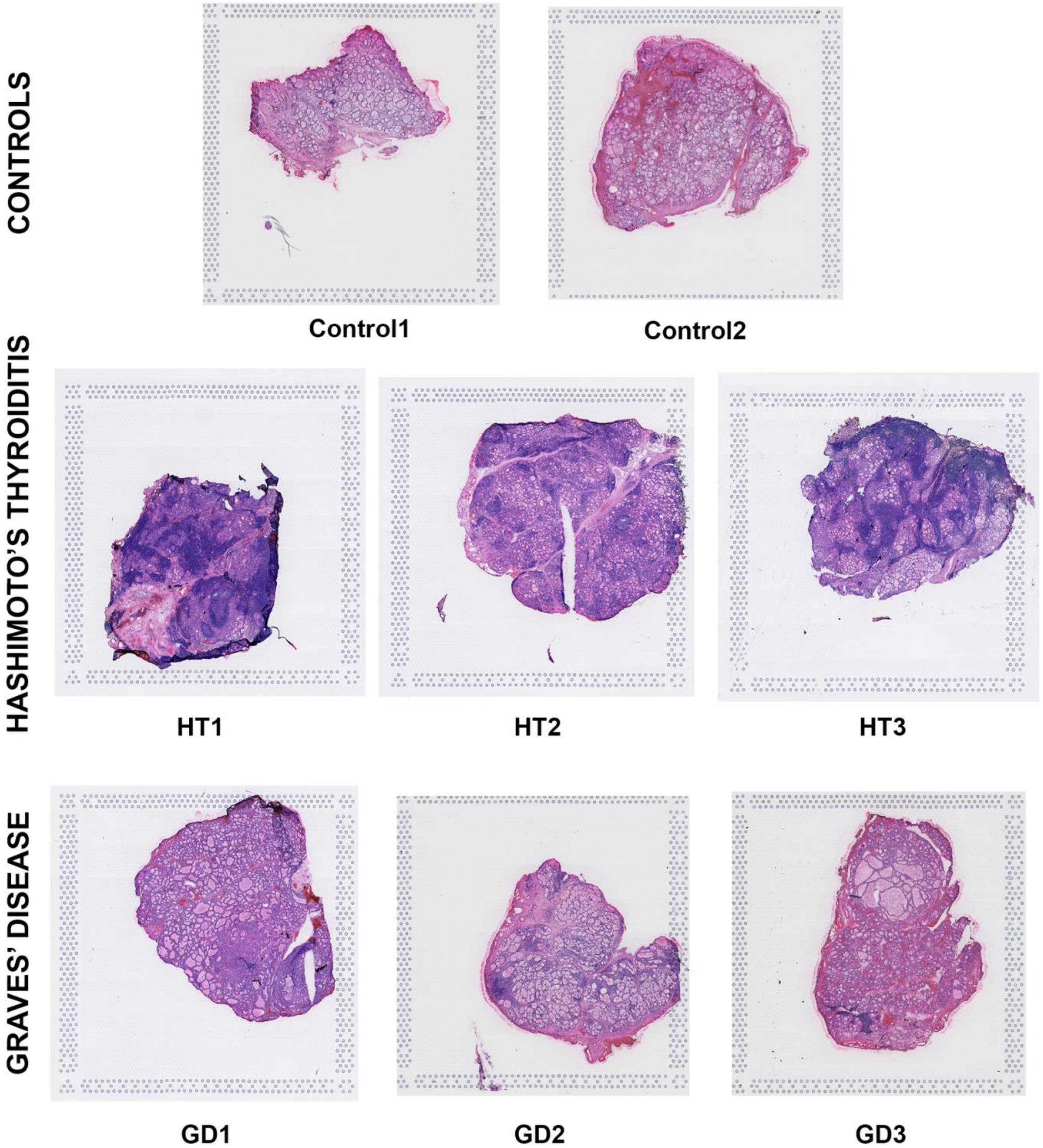

Supplementary Figure 1. Hematoxylin and eosin staining of three HT, three GD and two control samples. HT: Hashimoto's thyroiditis and GD: Graves' disease.

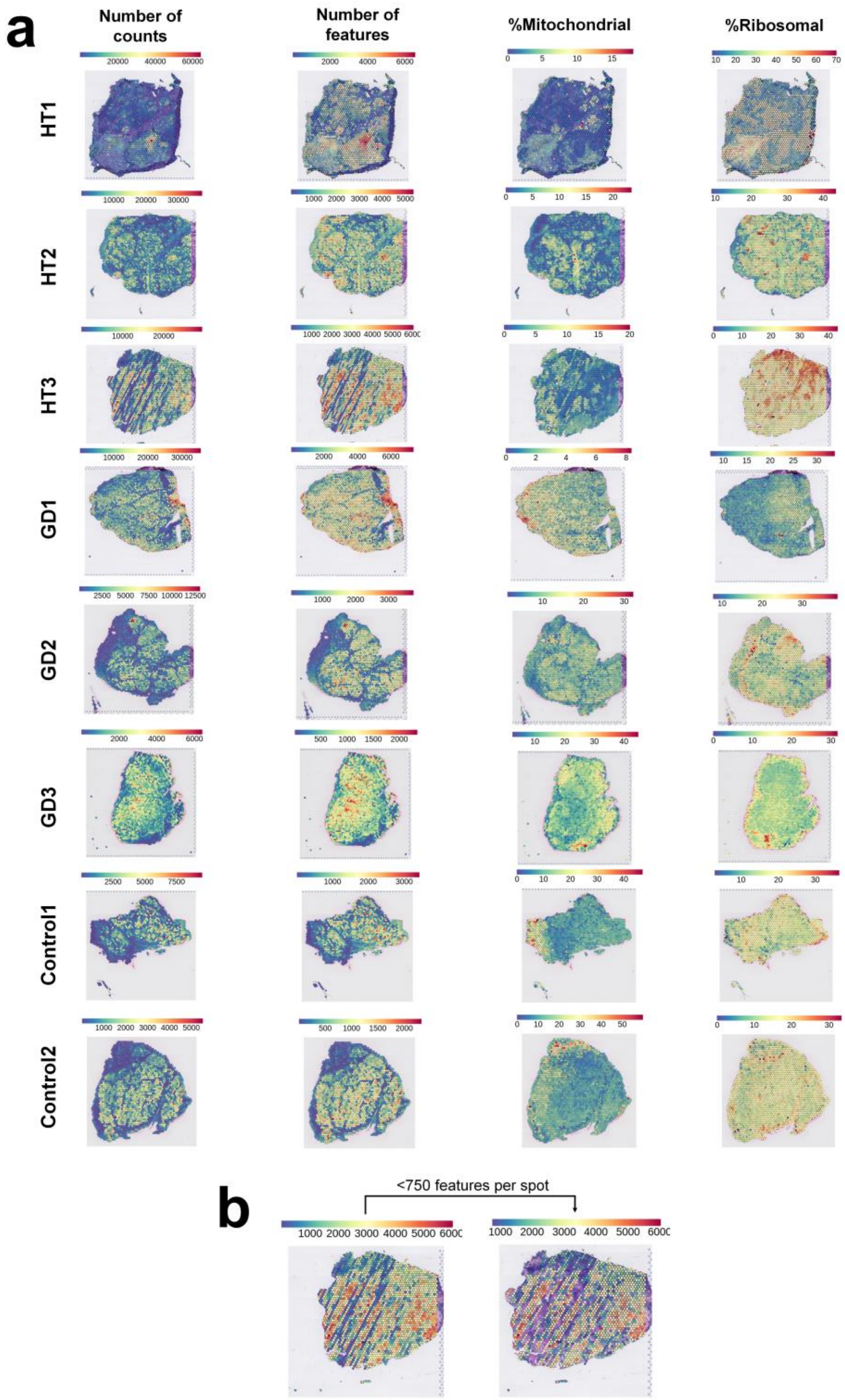

Supplementary Figure 2. **Quality control step of spatial transcriptomic data**. a) Quality control metrics of spatial transcriptomics samples showing number of counts and features, percentage of mitochondria and ribosomal protein genes. b) Removal of low quality spots matched with artificial scratches. HT: Hashimoto´s thyroiditis; GD: Graves´ disease.

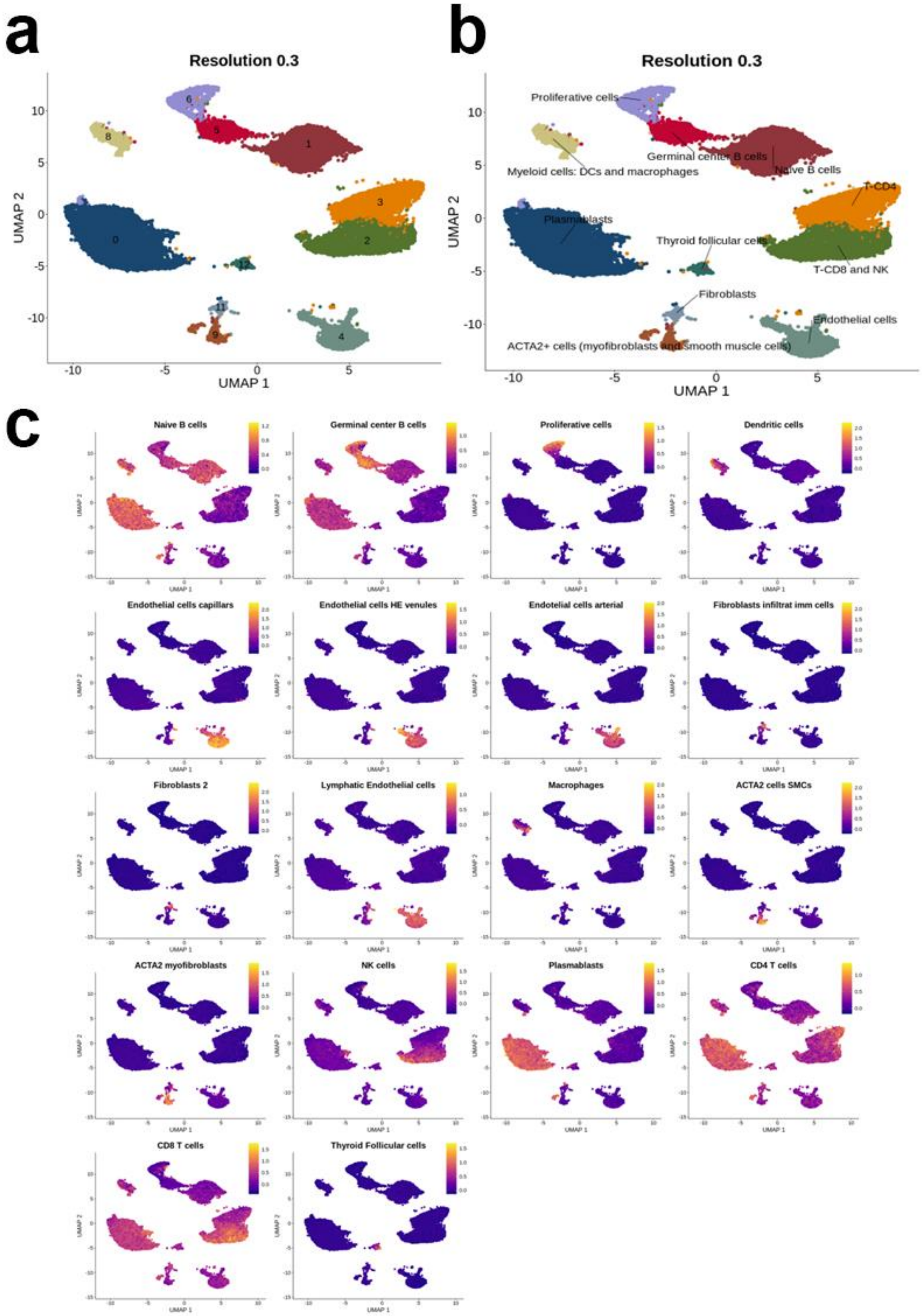

Supplementary Figure 3. **Analysis of public scRNAseq data from four HT patients.** Results of the integration of samples from HRA001684 data of patients with Hashimoto's thyroiditis. a) UMAP of Clustering at 0.3 resolution and b) cluster annotation. c) UMAP plot of each cell signature from HRA001684 repository.

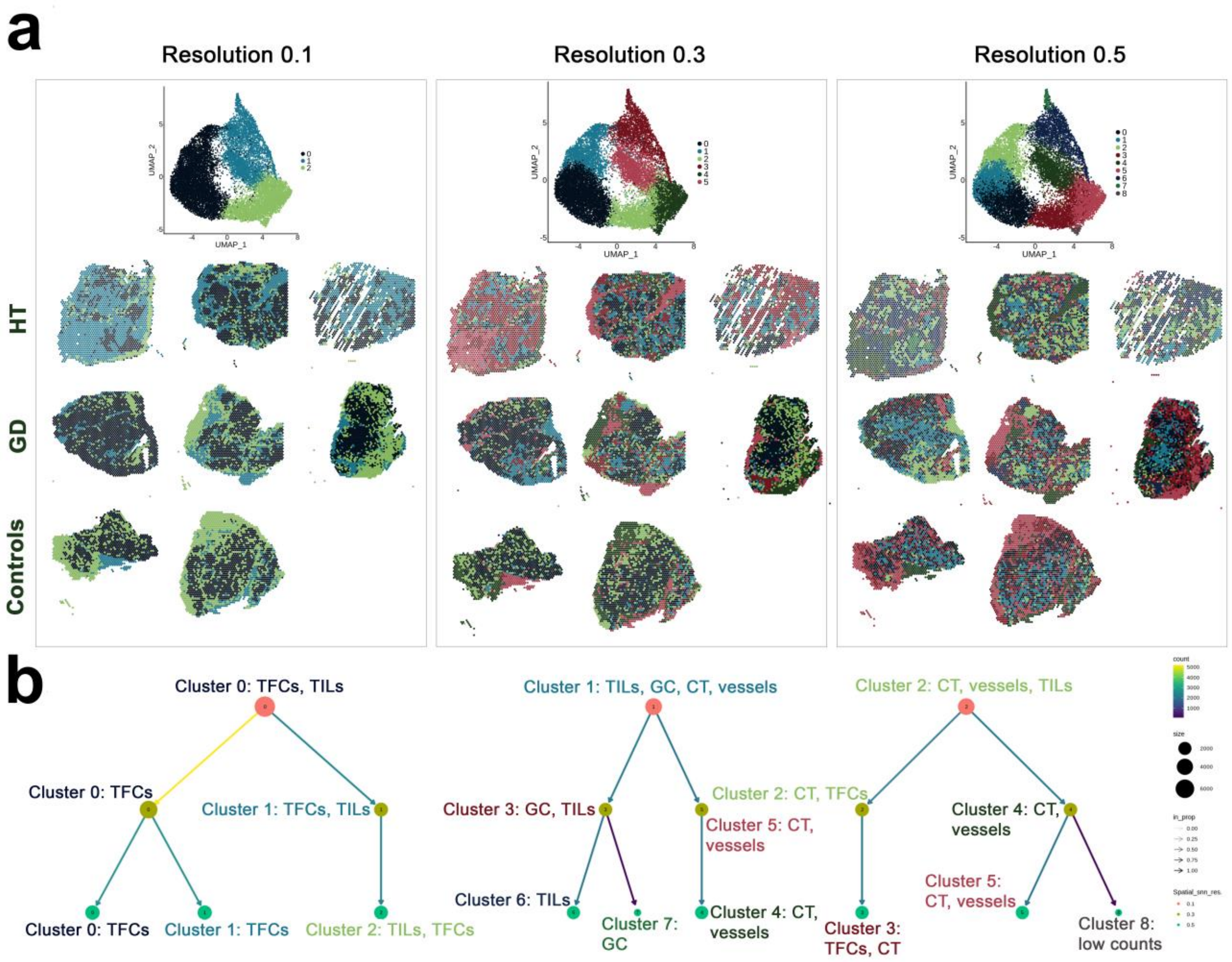

Supplementary Figure 4: Unsupervised analysis and clustering of the eight samples at 0.1, 0.3 and 0.5 resolutions. a) UMAP plots and spatial distribution of clusters across conditions (HT: Hashimoto's Thyroiditis; GD: Graves' disease; controls). b) Cluster tree to visualize the relationships between clusters at the different resolutions annotated with major cell types.

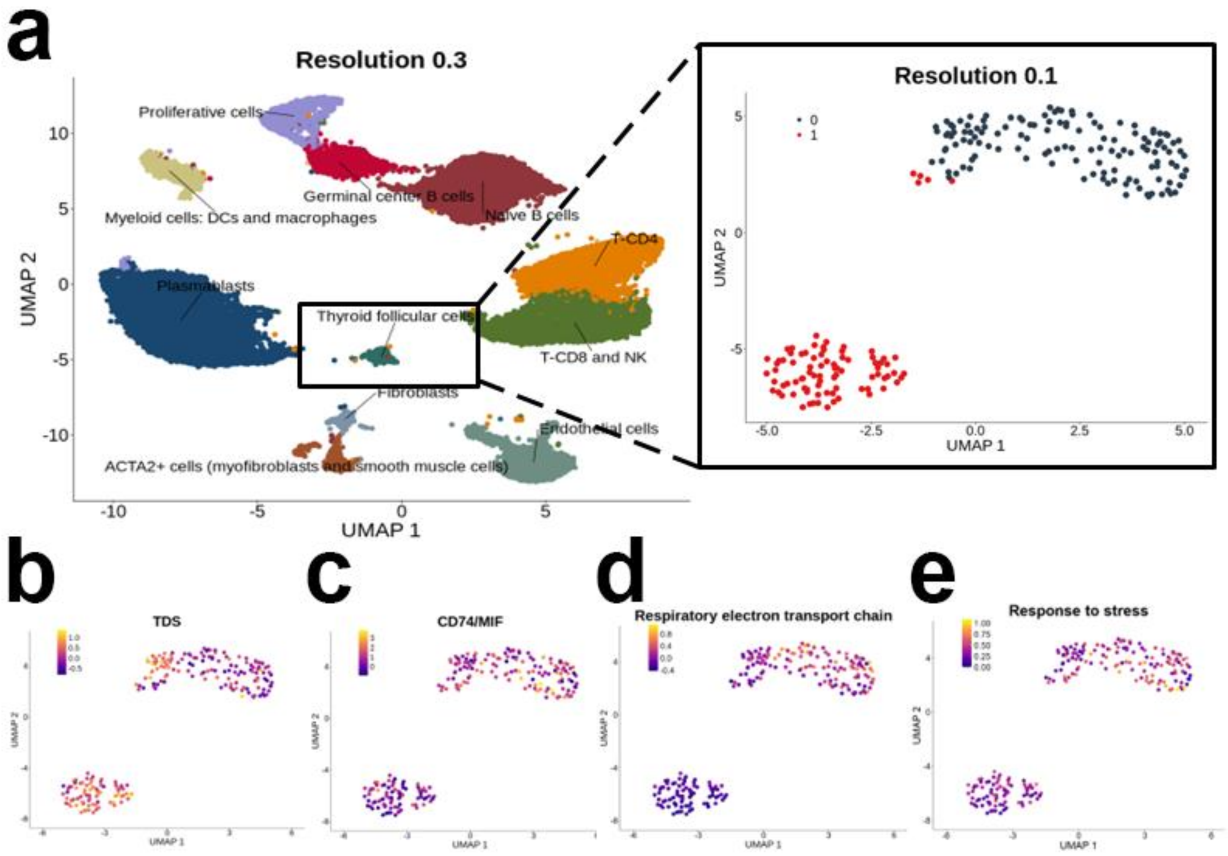

Supplementary Figure 5. Re-clustering of thyrocytes' cluster of scRNAseq data from public samples (HRA001684 experiment) of patients with Hashimoto's thyroiditis. a) UMAP of the re-clustering of thyrocytes at 0.1 resolution showing two different subpopulations. b) Thyrocyte differentiation score (TDS) c) CD74/MIF, d) Respiratory electron transport chain (ETC) and e) Response to stress signatures of the two clusters. UMAP: Uniform Manifold Approximation and Projection for Dimension Reduction.

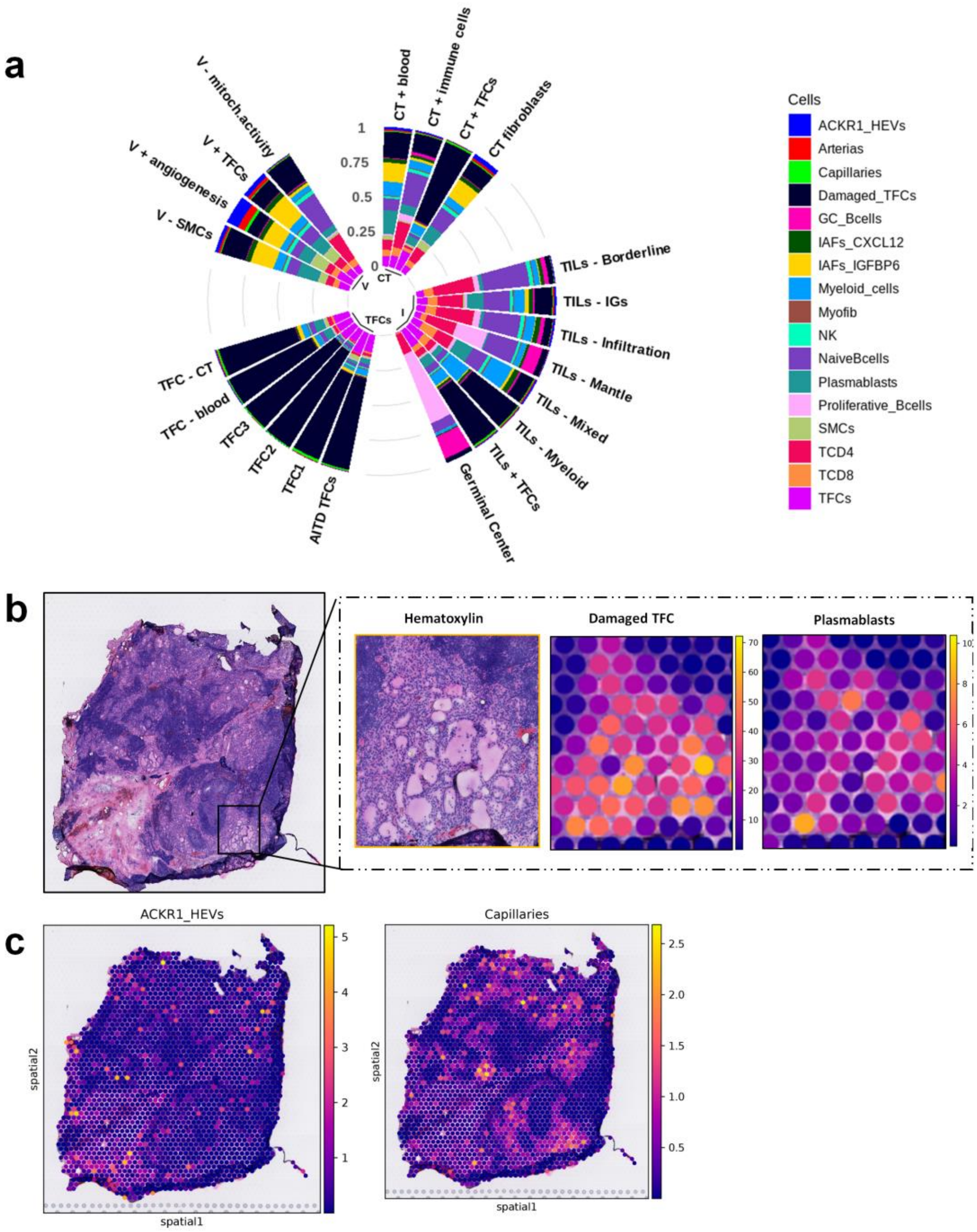

Supplementary Figure 6: Spots deconvolution using the cell signature from the analysis of scRNAseq data (HRA001684, Genome Sequence Archive (GSA). a) Total cell proportions found in the four regions analyzed (TFCs: Thyroid Follicular Cells; V: Vessels, CT: Connective Tissue and TILs: Thyroid Infiltrating lymphocytes). b) Hematoxylin and eosin and TFCs region detail showing the proportions of damaged TFCs and plasmablasts colocalizing at the same deconvoluted spots. c) ACKR1 and capillaries overview distributions. Side bars show the expected cell abundance.

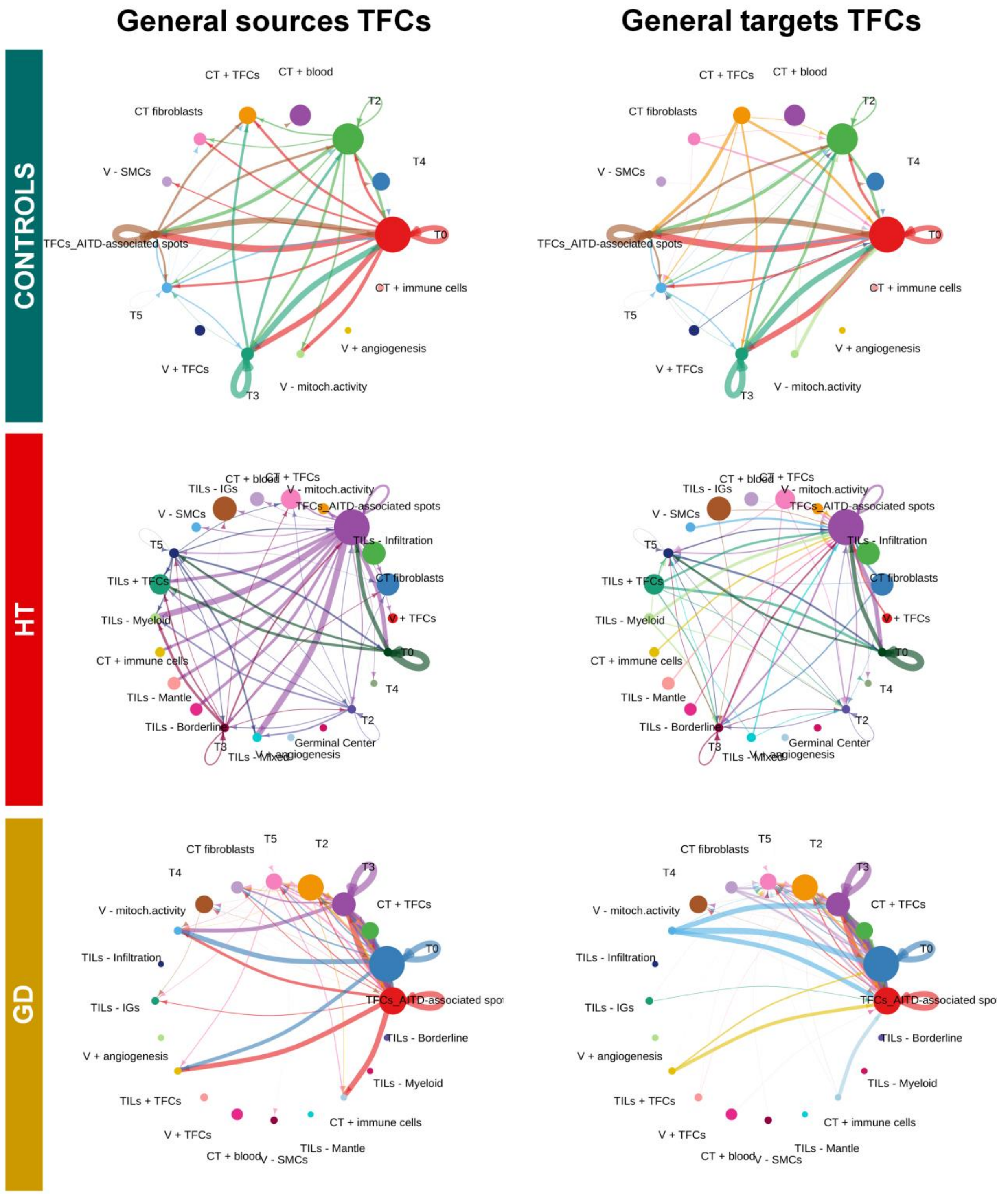

Supplementary Figure 7: Circle plots showing the intercellular communications of thyroid follicular cells (TFCs) to the other regions annotated in each condition. We separate TFCs regions as sources (left) and targets (right). Circle size correlates with the number of spots in the group and edges width represents the strength of the signal.

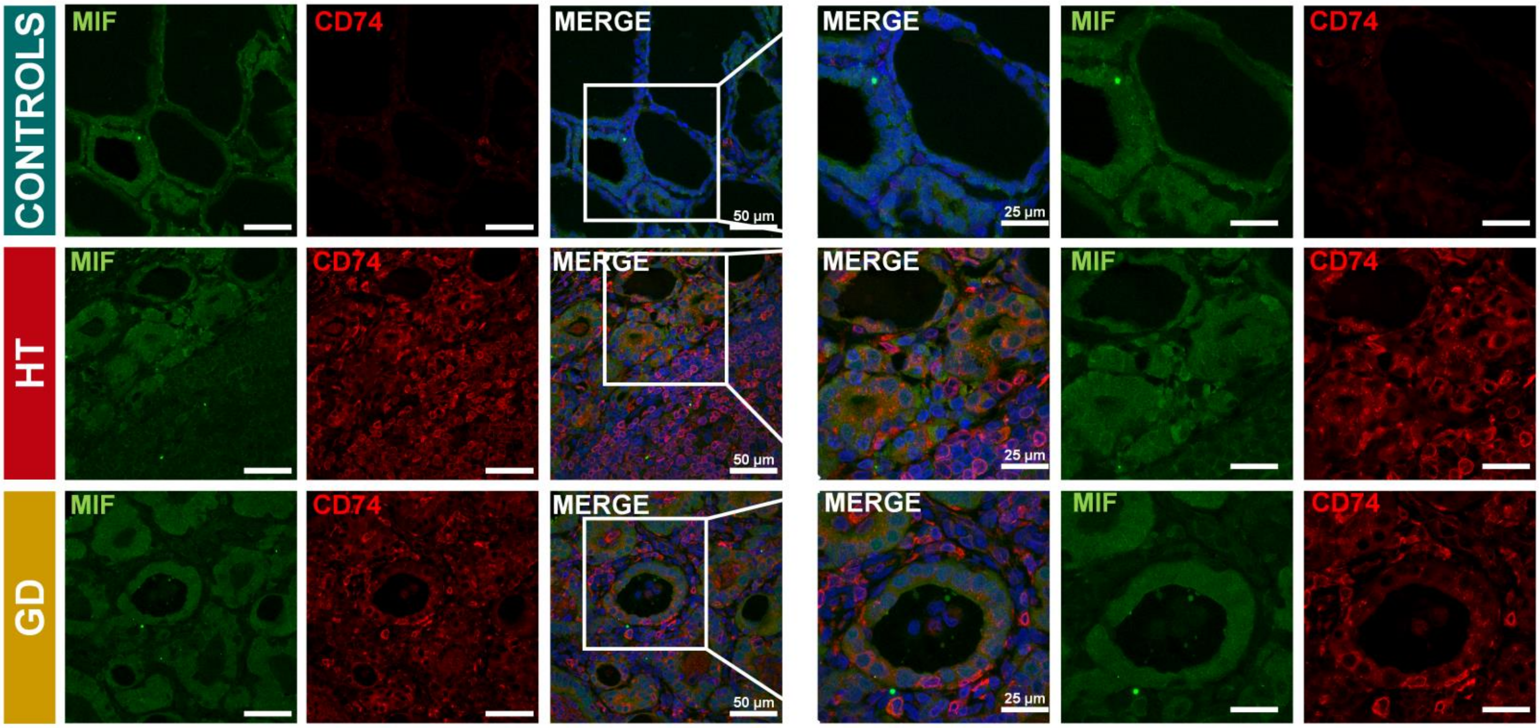

Supplementary Figure 8: CD74 and MIF immunofluorescence. Representative confocal immunofluorescence images, in healthy control, HT and GD tissue samples, of CD74 (red) and MIF (green). Nuclei are stained with DAPI (blue). Objective: 63X. Scale bar: 50 μm, zoom: 25 μm. Stainings were confirmed in at least seven biological replicates. HT: Hashimoto´s thyroiditis; GD: Graves´ disease.

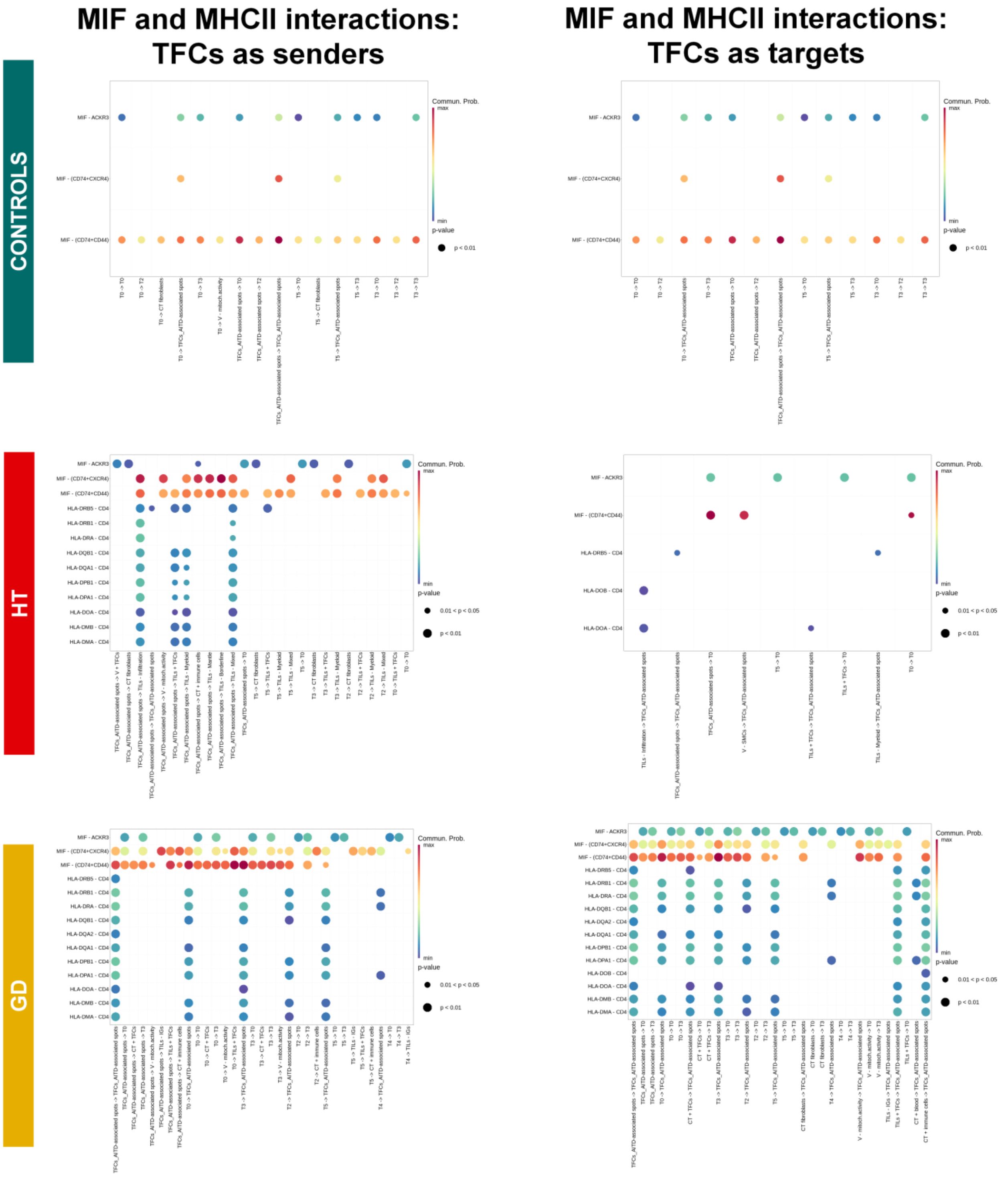

Supplementary Figure 9: **Overview of selected MIF and MHCII ligand-receptor interactions between TFCs and other regions annotated in each condition.** Circle size denotes p value and probability scores are indicated by colour.

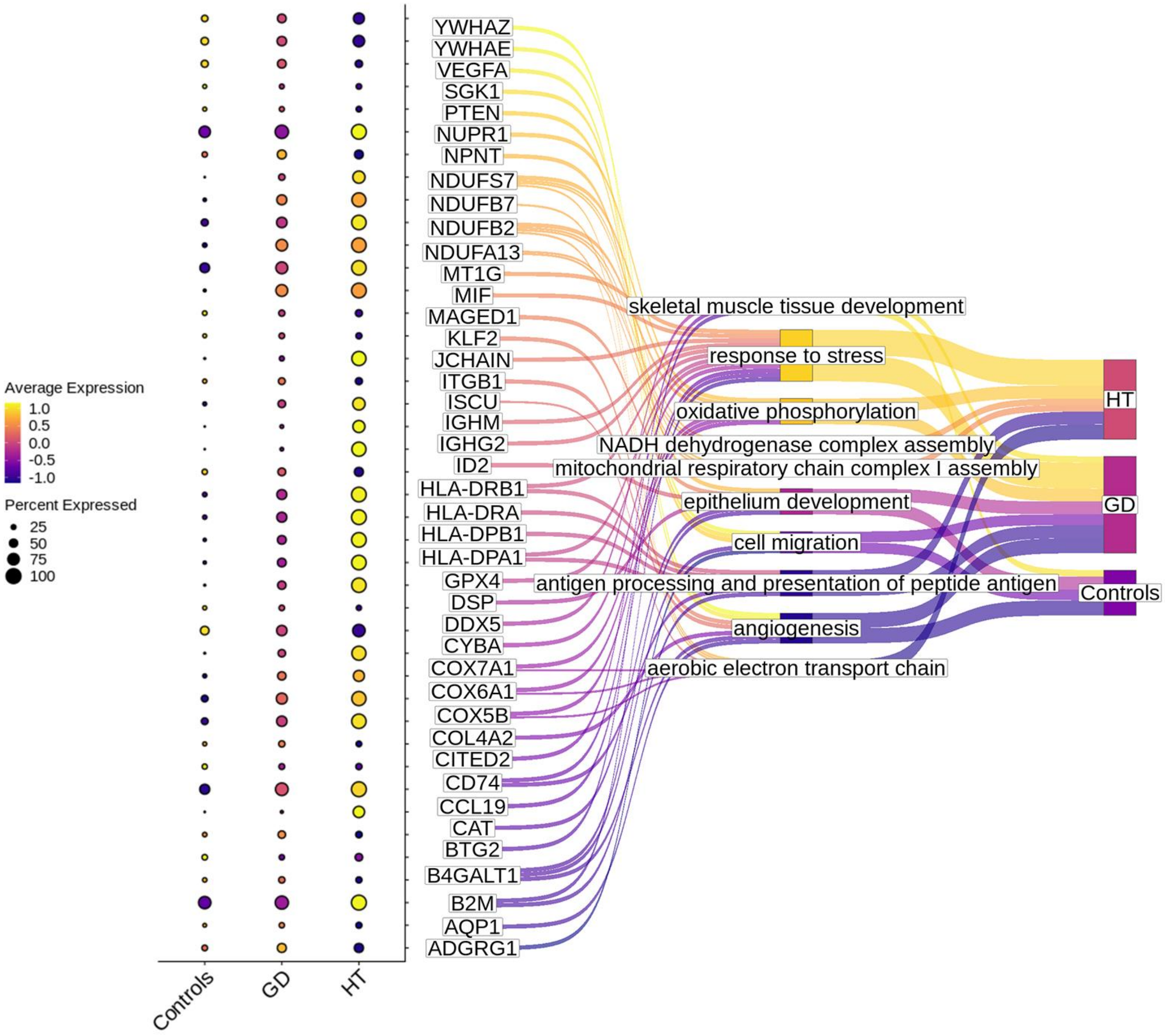

Supplementary Figure 10: Dot plot and sankey diagram of highlighted genes and pathways from the Thyroid follicular cells (TFCs) pseudobulk differential expression (DE) analysis. We performed the DE analysis between each condition, and its relation to HT, GD and controls. HT: Hashimoto´s thyroiditis; GD: Graves´ disease.

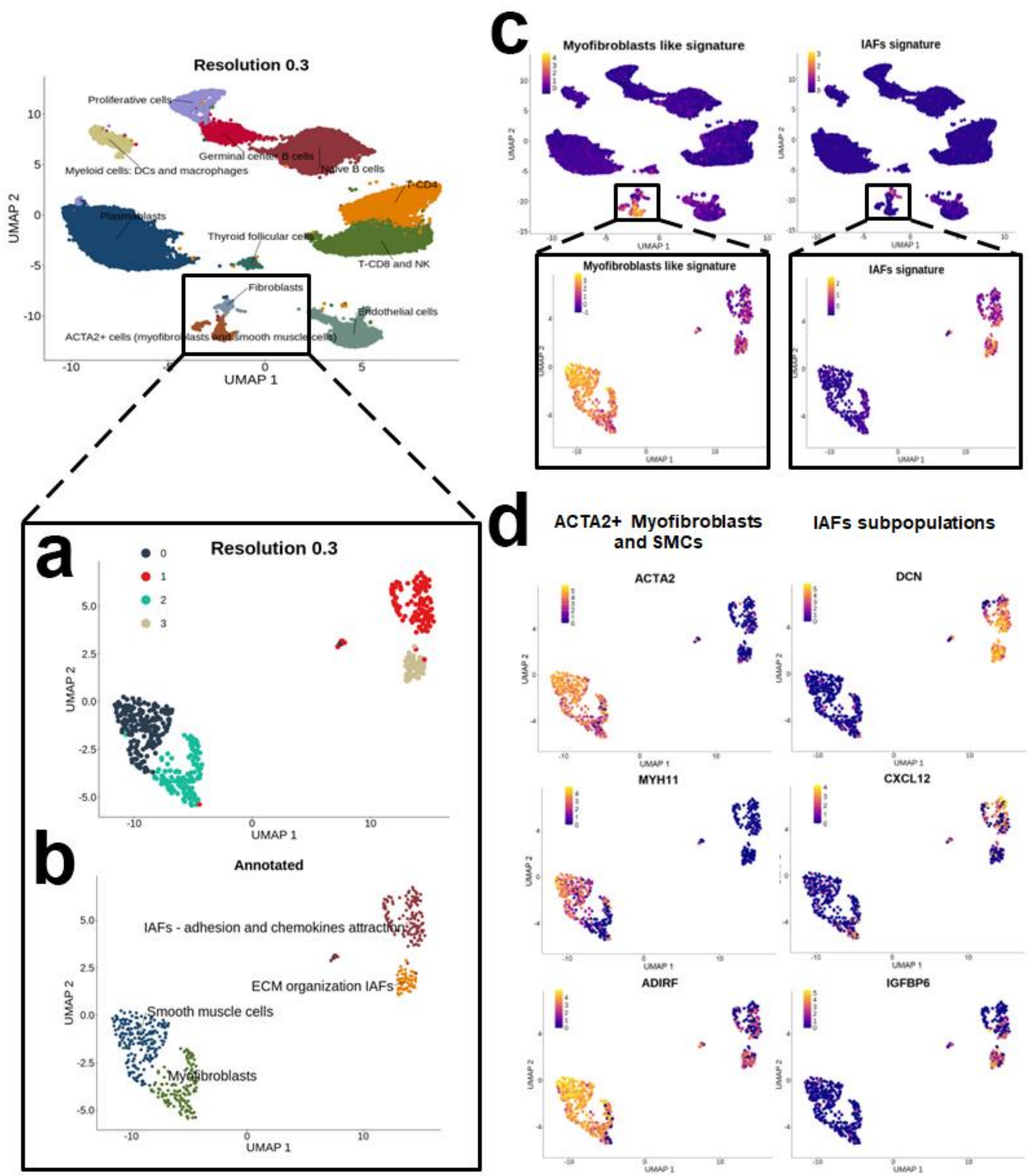

Supplementary Figure 11. Validation of myofibroblast-like and IAFs signatures using scRNAseq (HRA001684) of patients with Hashimoto's thyroiditis. a) UMAP of the re-clustering of ACTA2+ cells and fibroblasts at 0.3 resolution and b) cluster annotation. c) Correlation of both spatial transcriptomics signatures with the scRNAseq clusters. d) Main marker plotting. IAFs: inflammatory-associated fibroblasts; UMAP: Uniform Manifold Approximation and Projection for Dimension Reduction.

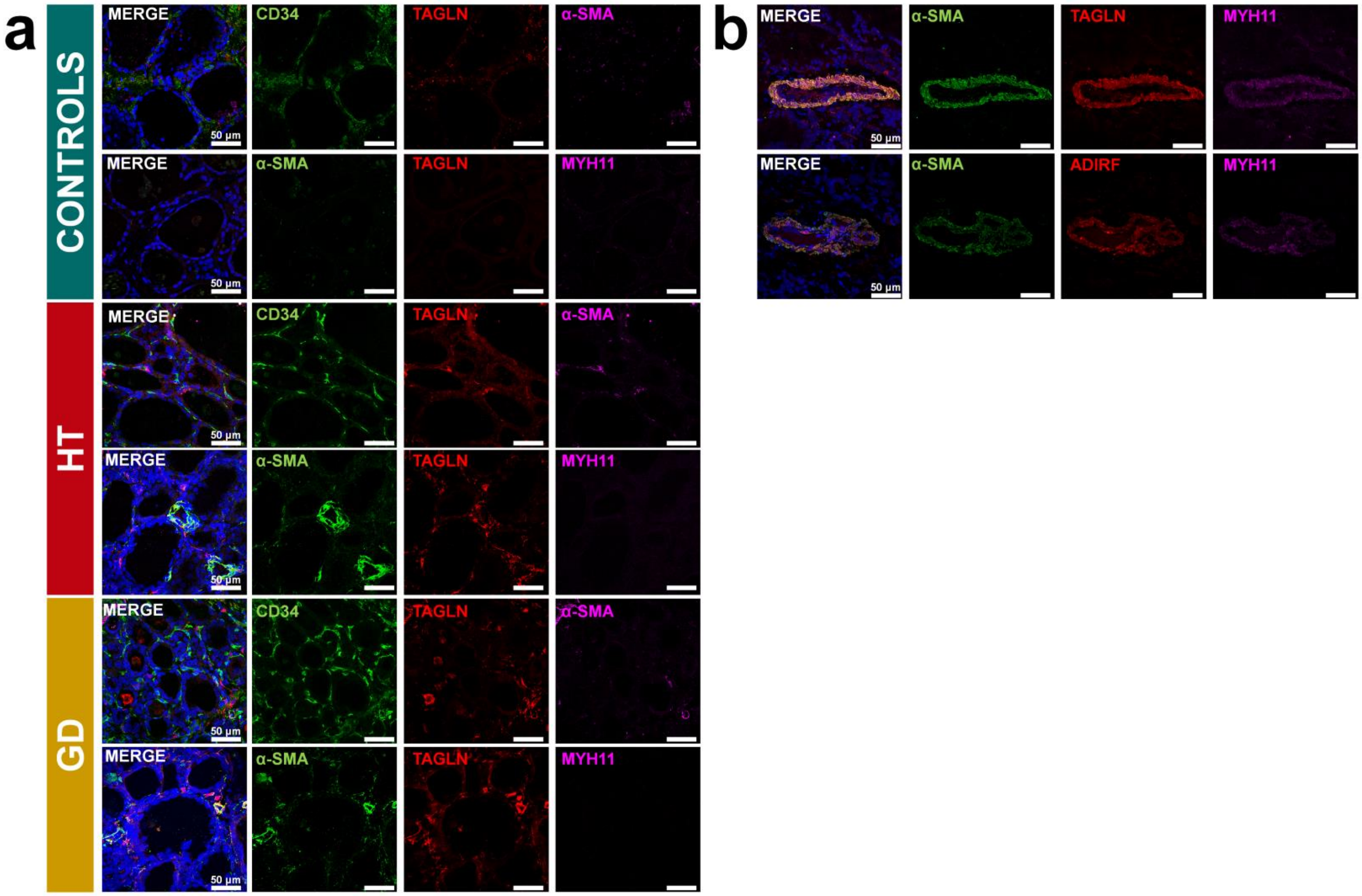

Supplementary Figure 12: **Immunofluorescence of TAGLN with other main myofibroblasts markers in AITD.** a) Representative confocal immunofluorescence images, in healthy controls, HT and GD tissue samples, of the myofibroblast markers α-SMA (magenta) and TAGLN (red) in combination with a mesenchymal marker, CD34 (green) or a smooth muscle cell marker, MYH11 (magenta). Nuclei are stained with DAPI (blue). Objective: 63X. Scale bar: 50 μm. b) Representative immunofluorescence of ADIRF/TAGLN (red), α-SMA (green) and MYH11 (magenta), for SMCs identification. Objective: 63X. Scale bar: 50 μm. Stainings were confirmed in at least seven biological replicates. HT: Hashimoto´s thyroiditis; GD: Graves´ disease; AITD: autoimmune thyroid diseases.

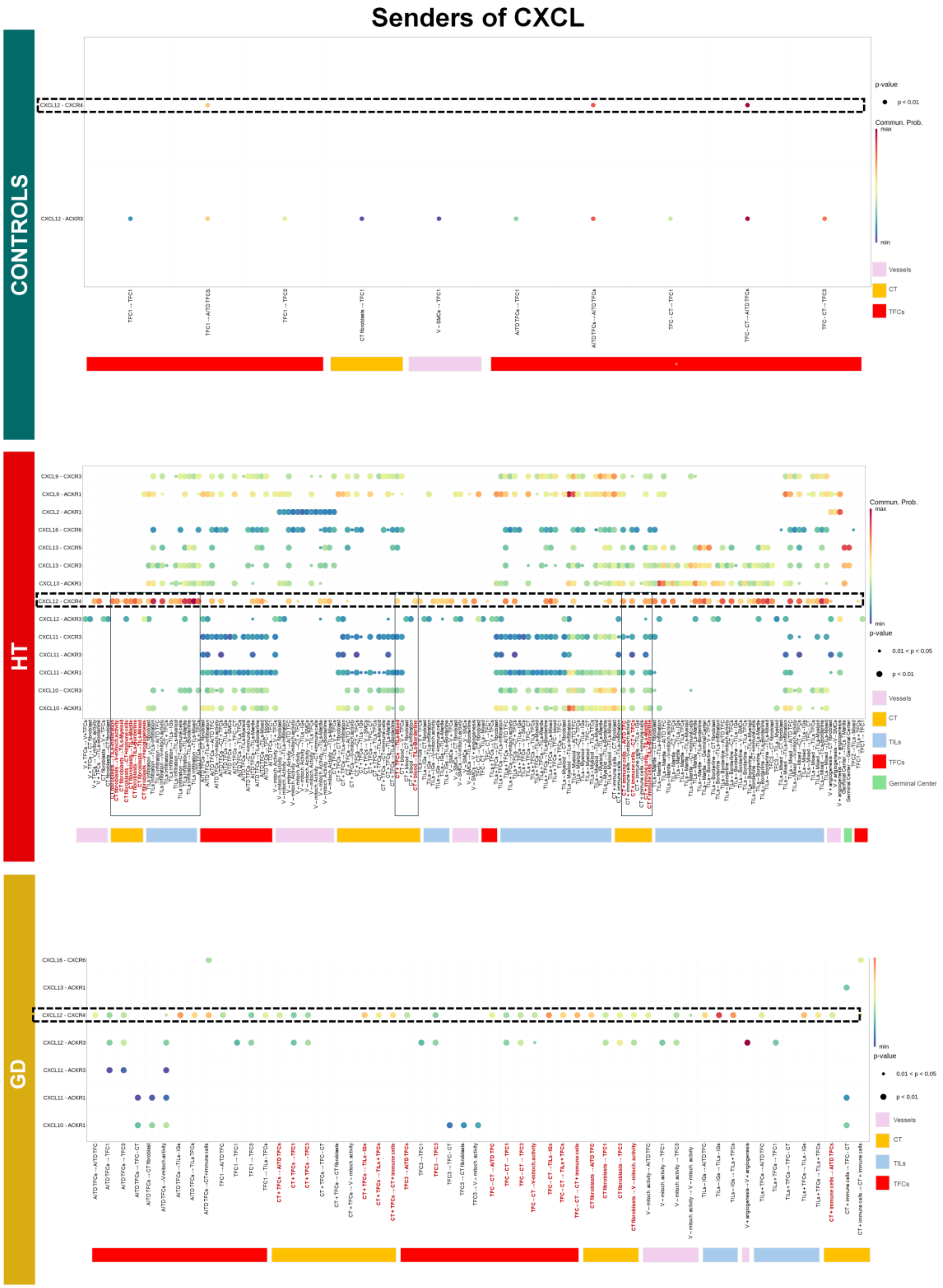

Supplementary Figure 13: Overview of selected CXCL ligand-receptor interactions among all the regions annotated in each condition. CXCL12- CXCR4 is highlighted with dashed lines. Circle size denotes p-value, and probability scores are indicated by colour.

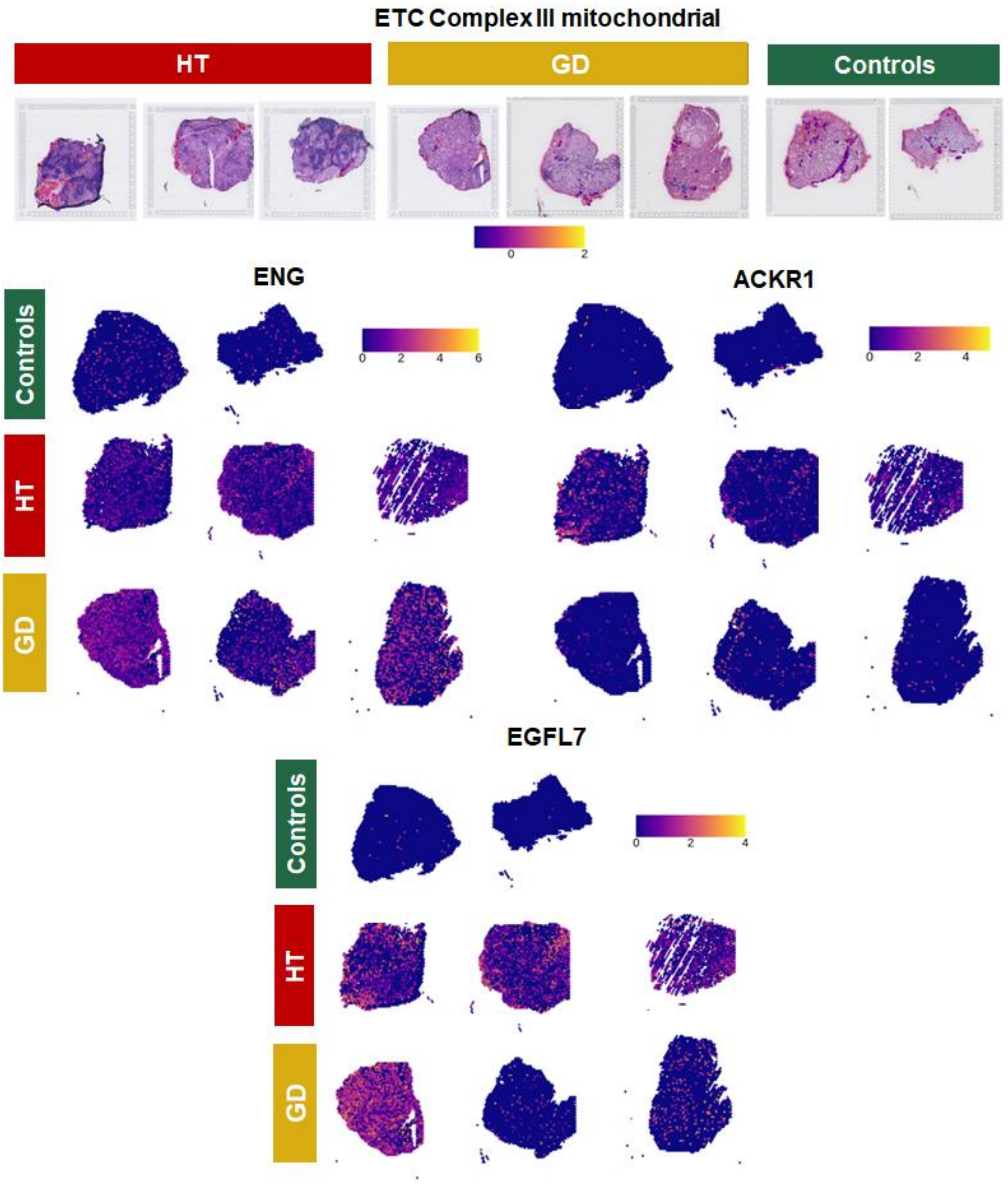

Supplementary Figure 14. Spatial distribution of endothelial markers associated to autoimmune thyroid diseases. ETC complex III mitochondrial genes (*UQCR11*, *UQCRQ* and *UQCRH*) in vessels areas. *ENG*, *ACKR1* and *EGFL7* expression distribution in HT, GD and control thyroid tissue. HT: Hashimoto´s thyroiditis; GD: Graves´ disease.

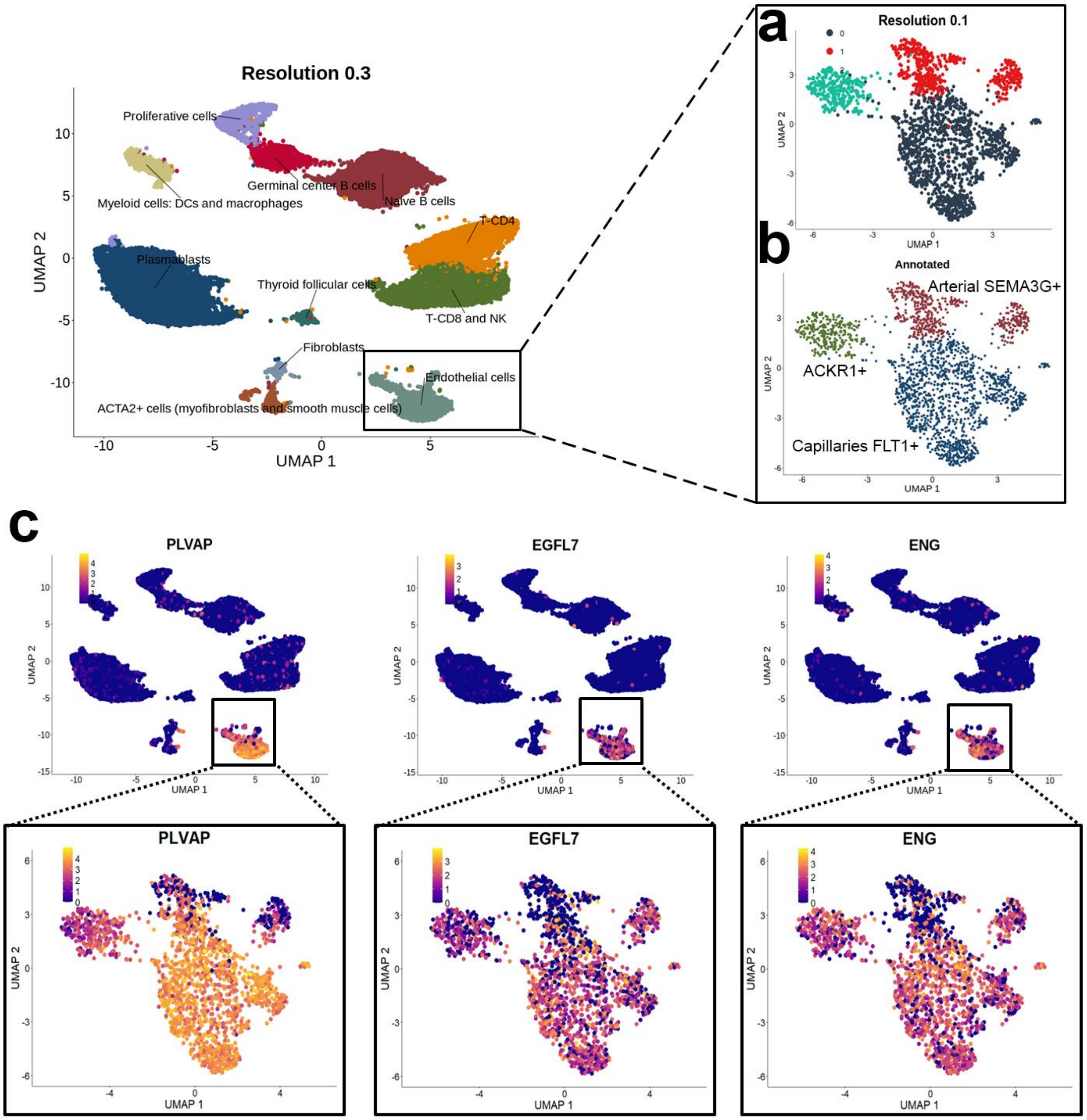

Supplementary Figure 15. Re-clustering of endothelial cells from patients with Hashimoto's thyroiditis using public scRNAseq data (HRA001684). a) UMAP of the re-clustering of the endothelium at 0.1 resolution. b) Cluster annotation. c) Remarkable expression of genes obtained from spatial transcriptomics (ST) data. HT: Hashimoto´s thyroiditis; GD: Graves´ disease; UMAP: Uniform Manifold Approximation and Projection for Dimension Reduction.

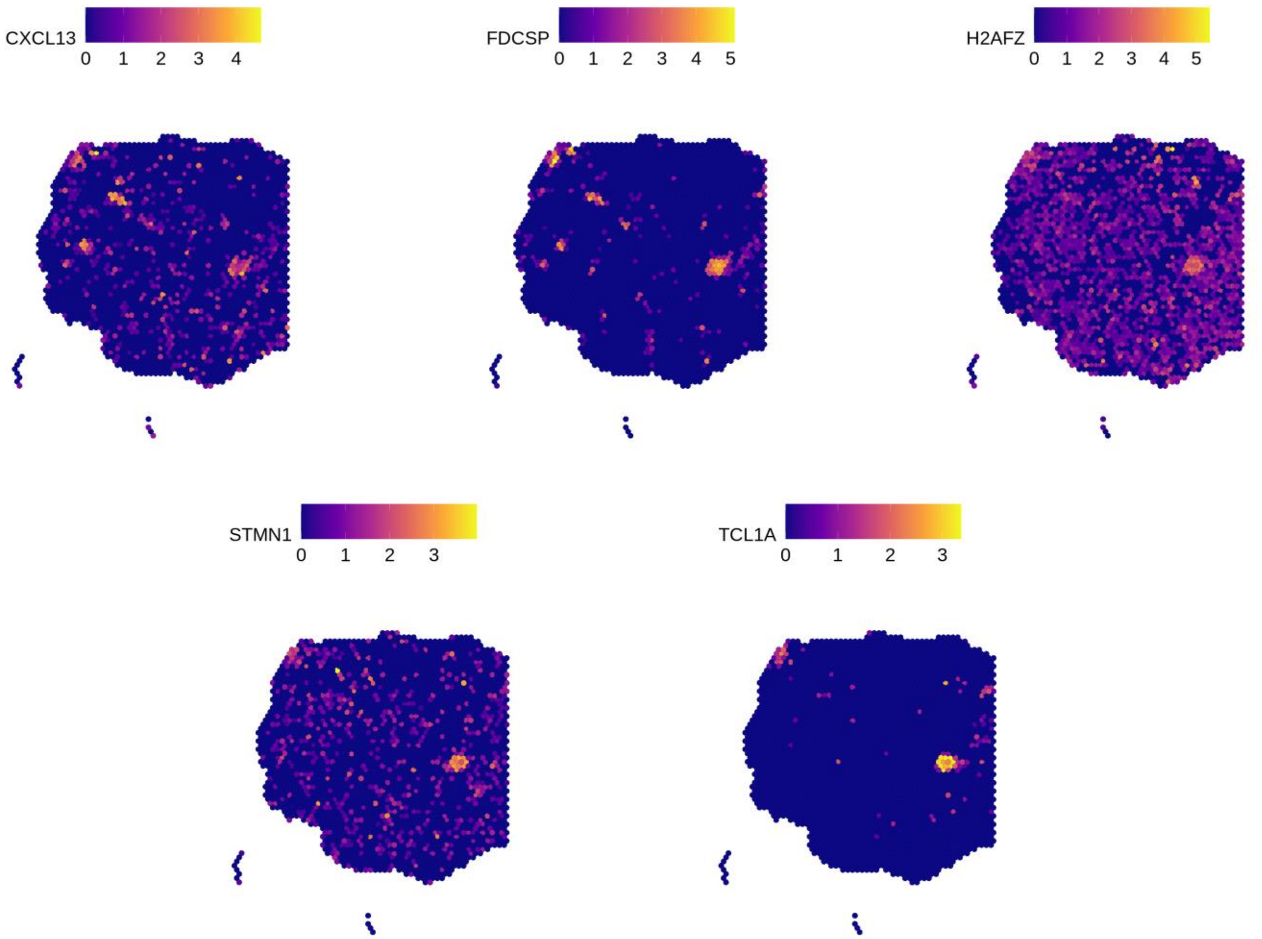

Supplementary Figure 16: Germinal center genes in a representative Hashimoto's thyroiditis (HT) sample. Visium sections from HT2 sample showing the top 5 genes specific from the germinal center region.

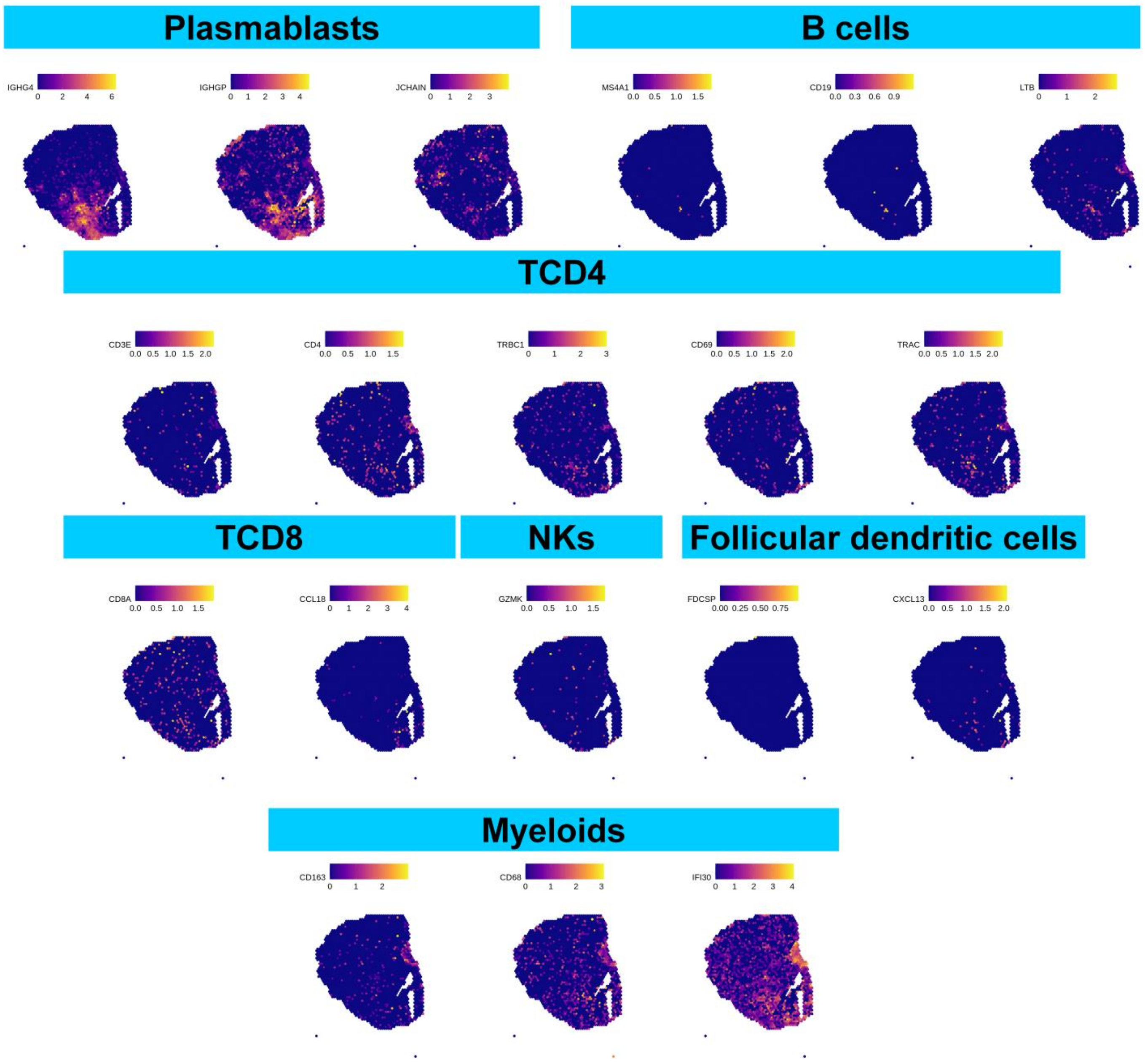

Supplementary Figure 17: Representative immune cells genes in Graves' disease (GD). Visium sections from GD1 sample showing the gene expression specific from each immune cell type lineage: plasmablasts, B cells, T CD4 cells, T CD8 cells, natural killer (NK) cells, follicular dendritic cells and myeloid cells.

Supplementary Table 1: Clinicopathological characteristics of spatial transcriptomics and tissue microarray cases

DIAGNOSTIC	SEX	AGE	T4	TSH	TPOAB	TGAB	TSHRAB	VISIUM SAMPLES	PATHOLOGICAL DIAGNOSIS	THYROID TREATMENT
CONTROL	M	53	NA	1,63	NA	NA	NA		Healthy tissue from contralateral papilar thyroid cancer	NO
CONTROL	F	35	1,58	3,44	<4	13	NA		Healthy tissue from a contralateral papilar thyroid microcarcinoma	NO
CONTROL	F	58	NA	NA	<4	<12	NA		Healthy tissue from a Hurtle adenoma	NO
CONTROL	M	62	NA	1,62	<4	<12	NA		Healthy tissue from a papilar thyroid microcarcinoma	NO
CONTROL	F	38	1,22	1,42	<4	<20	NA		Healthy tissue from an follicular thyroid adenoma	NO
CONTROL	M	58	NA	1,3	NA	NA	NA		Healthy tissue from a papilar thyroid microcarcinoma	NO
CONTROL	M	18	0,71	1,83	NA	NA	NA		Healthy tissue from a papilar thyroid cancer	NO
CONTROL	M	46	1,49	4	12	NA	NA		Healthy tissue from a papilar thyroid microcarcinoma	NO
CONTROL	F	55	NA	2,95	NA	NA	NA		Healthy tissue from a solitary adenoma	NO
CONTROL	F	68	NA	4,06	NA	NA	NA		Healthy tissue from an follicular thyroid adenoma	NO
CONTROL	F	50	1,18	0,02	<4	<12	NA		Healthy tissue from a papilar thyroid cancer	NO
CONTROL	F	57	NA	NA	NA	NA	NA		Healthy tissue from a Hurtle adenoma	NO
CONTROL	M	21	NA	NA	<4	<12	NA		Healthy tissue from a Hurtle adenoma	NO
CONTROL	M	71	NA	1,32	46	8,8	NA		Healthy tissue from a follicular adenoma	NO
CONTROL	F	42	NA	2,76	<4	<12	NA		Healthy tissue from a follicular adenoma	NO
CONTROL	F	64	NA	2,03	<4	<20	NA		Healthy tissue from a follicular adenoma	NO
CONTROL	F	65	NA	0,66	<4	<20	NA	C1	Healthy tissue from a papilar thyroid microcarcinoma	NO
CONTROL	F	54	NA	0,58	<4	<12	NA	C2	Healthy tissue from a papilar thyroid microcarcinoma	NO
CONTROL	F	64	NA	NA	NA	NA	NA		Healthy tissue from a multinodular goiter	NO
CONTROL	F	47	1,21	0,51	NA	NA	NA		Healthy tissue from a follicular adenoma	NO
CONTROL	M	61	NA	NA	NA	NA	NA		Healthy tissue from a contralateral multinodular goiter	NO
CONTROL	F	53	NA	3,92	NA	NA	NA		Healthy tissue from a contralateral multinodular goiter	NO
CONTROL	F	24	NA	NA	<4	<20	NA		Healthy tissue from a papilar thyroid microcarcinoma	NO
HT	F	59	NA	3,37	86	20	NA		Hashimoto's thyroiditis and thyroid nodule	NO
HT	F	74	1,36	4,75	4	31	NA		Hashimoto's thyroiditis and PTC	YES, levothyroxine
HT	F	62	NA	1,53	134	286	NA		Hashimoto's thyroiditis and PTC	NO
HT	M	53	NA	2,38	73	23	NA		Hashimoto's thyroiditis and PTC	YES, levothyroxine
HT	M	64	NA	2,26	335	193	NA		Hashimoto's thyroiditis and PTC	NO
HT	F	40	NA	4,03	134	70	NA		Hashimoto's thyroiditis and PTC	YES, levothyroxine
HT	F	32	NA	1,81	4	3069	NA		Hashimoto's thyroiditis and PTC	NO
HT	F	39	0,52	13,58	4	90	NA		Hashimoto's thyroiditis and PTC	NO
HT	M	34	1,72	4,68	647	511	NA	HT1	Hashimoto's thyroiditis and thyroid nodule	YES, levothyroxine
HT	F	51	NA	3,4	655	420	NA	HT2	Hashimoto's thyroiditis and thyroid nodule	NO
HT	F	61	1,31	5,28	NA	NA	NA	HT3	Hashimoto's thyroiditis	YES, levothyroxine
HT	F	79	NA	9	392	21	NA		Hashimoto's thyroiditis and PTC	YES, levothyroxine
HT	F	66	NA	6,18	259	12	NA		Hashimoto's thyroiditis and PTC	YES, levothyroxine
HT	F	17	NA	3,96	26	119	NA		Hashimoto's thyroiditis and PTC	NO
HT	F	75	NA	3,24	935	2801	NA		Hashimoto's thyroiditis and PTC	NO
HT	M	59	NA	3,58	77	12	NA		Hashimoto's thyroiditis and PTC	NO
HT	M	63	NA	0,78	775	3215	NA		Hashimoto's thyroiditis and thyroid nodule	NO
HT	F	48	NA	0,72	19	189	NA		Hashimoto's thyroiditis and PTC	NO
HT	F	34	0,79	5,42	215	6318	NA		Hashimoto's thyroiditis and thyroid nodule	NO
HT	F	34	NA	NA	190	454	NA		Hashimoto's thyroiditis and Adenoma	YES, levothyroxine
HT	M	47	0,7	7	2519	>20000	NA		Hashimoto's thyroiditis	NO
HT	F	65	NA	2,5	<4	17081	NA		Hashimoto's thyroiditis	NO
HT	F	24	1,03	1,6	583	293	11,24		Hashimoto's thyroiditis and thyroid nodule	NO
HT	F	69	0,84	5,82	222	<20	NA		Hashimoto's thyroiditis and thyroid nodule	YES, levothyroxine
HT	F	56	NA	2,59	<20	556	NA		Hashimoto's thyroiditis and thyroid nodule	YES, levothyroxine
HT	F	54	1,1	0,48	2000	67	NA		Hashimoto's thyroiditis and thyroid nodule	NO
HT	F	35	NA	2,44	1321	1458	4,6		Hashimoto's thyroiditis	NO
HT	F	47	NA	2,7	312	140	NA		Hashimoto's thyroiditis	NO
HT	F	55	NA	1,06	NA	NA	NA		Hashimoto's thyroiditis	YES, levothyroxine
GD	F	70	0,73	13,52	NA	NA	3,05	GD3	Graves disease	Anti-Thyroid treatment
GD	F	46	2,06	0,01	16	117	1,11	GD2	Graves disease	Anti-Thyroid treatment
GD	F	41	0,96	0,01	NA	NA	2,52		Graves disease	Anti-Thyroid treatment
GD	F	44	1,33	2,01	NA	471	1,97		Graves disease	Anti-Thyroid treatment
GD	M	54	1,06	4,34	NA	NA	2,96		Graves disease	Anti-Thyroid treatment
GD	F	21	1,57	0,01	964	181	31,8		Graves disease	Anti-Thyroid treatment
GD	F	39	0,76	0,15	NA	NA	4,54		Graves disease	Anti-Thyroid treatment
GD	F	22	1,35	0,01	NA	NA	23,76	GD1	Graves disease	Anti-Thyroid treatment
GD	F	20	0,55	10,36	175	23	3,1		Graves disease	Anti-Thyroid treatment
GD	F	54	1,02	0,01	NA	NA	300		Graves disease	Anti-Thyroid treatment
GD	F	44	1,65	0,01	NA	NA	5,89		Graves disease	Anti-Thyroid treatment
GD	F	62	1,03	5,73	223	95	7,25		Graves disease	Anti-Thyroid treatment
GD	F	56	1,33	1,44	4	12	1,31		Graves disease	Anti-Thyroid treatment
GD	F	74	3,77	2,94	NA	<20	6,35		Graves disease	Anti-Thyroid treatment
GD	F	40	5,09	0,01	NA	NA	8,39		Graves disease	Anti-Thyroid treatment
GD	F	58	0,95	7,42	NA	NA	2,1		Graves disease	Anti-Thyroid treatment
GD	F	38	1,3	0	NA	0,01	6,27		Graves disease	Anti-Thyroid treatment
GD	F	47	0,65	4,85	NA	NA	>0.7		Graves disease	Anti-Thyroid treatment
GD	F	41	1	0,05	NA	NA	26,8		Graves disease	Anti-Thyroid treatment
GD	F	37	16,99	0,01	NA	NA	2,6		Graves disease	Anti-Thyroid treatment
GD	F	55	1,3	0,01	20	649	14,84		Graves disease	Anti-Thyroid treatment
GD	M	36	1,61	0,01	NA	NA	1,71		Graves disease	Anti-Thyroid treatment
GD	F	23	1,4	0,01	901	732	2,4		Graves disease	Anti-Thyroid treatment
GD	F	28	1,41	0,6	102	20	NA		Graves disease	Anti-Thyroid treatment

F/M = female/male; TSH, thyrotropin (Normal range=0.27-4.20 mU/ml); T4, thyroxine (Normal range=0.93-1.7 ng/dL); Tg-Ab, anti-thyroglobulin antibody (Negative<344 IU/mL for those samples diagnosed before February 2018 and Negative <60 IU/mL for the rest); TPO-Ab, anti-thyroid peroxidase antibody (Negative<100 IU/mL for those samples diagnosed before February 2018 and Negative <35 IU/mL for the rest) TSHR-Ab, anti-thyrotropin receptor antibody (Negative<0.7 U/L)

Supplementary Table 2: Antibodies used in the study

Target	Cell subpopulation	Host	Company	Reference (Catalogue number)	Antibody ID	PH Retrieval	Dilution
CD74	TFCs	Rabbit	Abcam. Cambridge, UK	ab64772	AB_1658627	9	1/150
MIF	TFCs	Mouse	Thermo Fisher Scientific. Waltham, MA, USA	MA1-20881	AB_2144072	9	1/100
CD34	Fibroblasts	Mouse	Agilent. Santa Clara, CA, USA	M7165	AB_2063006	9	1/50
ADIRF	Myofibroblasts	Rabbit	Sigma Aldrich. Saint Louis, MO, USA	HPA026810	AB_2672003	9	1/100
TAGLN	Myofibroblasts	Rabbit	Abcam. Cambridge, UK	ab155272	AB_2637003	9	1/100
ALPHA-SMA	Myofibroblasts/SMCs	Goat	Thermo Fisher Scientific. Waltham, MA, USA	PA5-18292	AB_10980764	9	1/150
MYH11	SMCs	Mouse	Thermo Fisher Scientific. Waltham, MA, USA	MA5-11971	AB_11004268	9	1/50
DCN	Generic IAFs	Rabbit	Abcam. Cambridge, UK	ab175404	AB_2890261	9	1/50
CXCL12	Chemoattractant IAFs	Rabbit	Thermo Fisher Scientific. Waltham, MA, USA	PA5-89116	AB_2805365	9	1/100
IGFBP6	ECM organizers IAFs	Mouse	Proteintech. Rosemont, IL, USA	67567-1-Ig	AB_2882781	9	1/50
PLVAP	Fenestrated vessels	Rabbit	Thermo Fisher Scientific. Waltham, MA, USA	PA5-51698	AB_2645688	9	1/250
