# Supplementary material for: Unraveling the molecular architecture of autoimmune thyroid diseases at spatial resolution
Source: Nat Commun. 2024 Jul 13;15:5895. doi: 10.1038/s41467-024-50192-5 (PMC11246508; doi:10.1038/s41467-024-50192-5)
Supplement: Supplementary file 3 — Description of Additional Supplementary Files [file 41467_2024_50192_MOESM3_ESM.pdf]

Description of Additional Supplementary Files

Supplementary Data 1: Top marker genes of the eight regions identified in histologic annotation of the thyroid.

Supplementary Data 2: Marker genes of the different molecular signatures and scores used in the study.

Supplementary Data 3: Marker genes of clusters from thyroid follicular cell regions.

Supplementary Data 4: Results of differential expression gene analysis between cluster T1 and the others from TFCs regions.

Supplementary Data 5: Marker genes of clusters from connective tissue regions.

Supplementary Data 6: Marker genes of clusters from vessel-annotated spots.

Supplementary Data 7: Marker genes of clusters from thyroid infiltrating lymphocytes regions.

Supplementary Data 8: Marker genes of clusters obtained from I0 TILS reclustering.

Supplementary Data 9: Results of differential expression gene analysis of TILs pseudobulk between HT and GD.
