# Supplementary material for: Unraveling the molecular architecture of autoimmune thyroid diseases at spatial resolution
Source: Nat Commun. 2024 Jul 13;15:5895. doi: 10.1038/s41467-024-50192-5 (PMC11246508; doi:10.1038/s41467-024-50192-5)
Supplement: Supplementary file 13 — Reporting Summary [file 41467_2024_50192_MOESM13_ESM.pdf]

nature portfolio

Corresponding author(s): Rebeca Martínez Hernández

Last updated by author(s): 2024/05/08

Reporting Summary

Nature Portfolio wishes to improve the reproducibility of the work that we publish. This form provides structure for consistency and transparency in reporting. For further information on Nature Portfolio policies, see our Editorial Policies and the Editorial Policy Checklist.

Statistics

For all statistical analyses, confirm that the following items are present in the figure legend, table legend, main text, or Methods section.

n/a	Confirmed	
☐	☒	The exact sample size (n) for each experimental group/condition, given as a discrete number and unit of measurement
☐	☒	A statement on whether measurements were taken from distinct samples or whether the same sample was measured repeatedly
☐	☒	The statistical test(s) used AND whether they are one- or two-sided<br>*Only common tests should be described solely by name; describe more complex techniques in the Methods section.*
☒	☐	A description of all covariates tested
☐	☒	A description of any assumptions or corrections, such as tests of normality and adjustment for multiple comparisons
☐	☒	A full description of the statistical parameters including central tendency (e.g. means) or other basic estimates (e.g. regression coefficient) AND variation (e.g. standard deviation) or associated estimates of uncertainty (e.g. confidence intervals)
☐	☒	For null hypothesis testing, the test statistic (e.g. F, t, r) with confidence intervals, effect sizes, degrees of freedom and P value noted<br>*Give P values as exact values whenever suitable.*
☒	☐	For Bayesian analysis, information on the choice of priors and Markov chain Monte Carlo settings
☐	☒	For hierarchical and complex designs, identification of the appropriate level for tests and full reporting of outcomes
☐	☒	Estimates of effect sizes (e.g. Cohen's d, Pearson's r), indicating how they were calculated

Our web collection on statistics for biologists contains articles on many of the points above.

Software and code

Policy information about availability of computer code

Data collection

High quality images and raw ST data were processed using SpaceRanger 10x Genomics software version 1.2.2 and mapped to the human genome reference GRCh38.

Data analysis

Data preprocessing
High quality images and raw ST data were processed using SpaceRanger 10x Genomics software version 1.2.2 and mapped to the human genome reference GRCh38. Quality control was evaluated using R (version 4.0.3) and Seurat package29. To define the sequencing quality scores of each sample we established the recommended threshold and quality control metrics from 10x Genomics. Then, we evaluated the spatial plotting of the number of counts, number of features and percentage of ribosomal and mitochondrial genes. Low quality spots were removed.
(Code available in https://github.com/endonutriHUPR/AITD_SpatialTranscriptomics.git).

Histology-based region selection
Hematoxylin and eosin (H&E) thyroid images (Supplementary Figure 1) were evaluated and annotated manually by two independent trained pathologists using Loupe browser software 5.0 interface (10x Genomics). Spots included in destructed or broken tissue, low quality control metrics or tissue-fold artifacts were excluded from any further analysis (Supplementary Figure 2). Samples were annotated for specific cell tissue populations according to their histology and then validated by their molecular/transcriptomic profile.

Normalization, dimensional reduction, integration and batch effect correction
Data logarithmic normalization (2000 variable features) of the eight samples was performed using NormalizeData() from Seurat package (version 4.2.0)29 in R (version 4.0.3). 3000 variable features were chosen to merge and scale all samples together. To correct batch effects of

"samples" and "experimental day", we performed a Principal Component Analysis (PCA) with the top 30 dimensions and a Harmony integration30.

Clustering and differential expression within cell populations
To investigate specific cell states and map the heterogeneity of each compartment (TFCs, connective tissue, vessels and immune infiltration), we addressed the analysis as follows: 1) from the Harmony integration (previously assessing the number of dimensions by the elbow method), we applied FindClusters() function at 0.1, 0.3 and 0.5 resolutions to identify main clusters followed by Uniform Manifold Approximation and Projection (UMAP) generation and re-clustering when required. Then, we identified cluster-defined genes by FindAllMarkers() function with default options. 2) A pseudo-bulk differential expression (DE) analysis of each compartment through Wilcoxon algorithm between conditions. 3) A general DE considering the whole tissue between conditions. This step was also implemented in Seurat package (version 4.2.0)29, in R (version 4.0.3).

Biological enrichment analysis
We used clusterProfiler package31 for gene set enrichment analysis (GSEA)32 to identify gene sets and the different pathways disturbed in each tissue cell population. In addition, we explored specific pathways with KEGG33 and Reactome34 databases. AddModuleScore() from Seurat was used to identify, quantify and plot remarkable signatures. Bioinformatic analysis code is available at GitHub: https://github.com/endonutriHUPR/AITD_SpatialTranscriptomics.git

Single cell analysis
Published single-cell data from the thyroid of 4 HT patients27, accession number HRA001684 for the Genome Sequence Archive (GSA), were analyzed to validate cell populations obtained by ST results. DoubletFinder R package35 was applied in each sample to filter doublets. Then, we followed the authors' guidelines using Seurat (version 4.2.0)29 workflow and Harmony30 to correct the samples' batch effects. Cell re-clustering was performed by SplitObject(), followed by a logarithmic scale normalization and keeping the 2000 variable features. Then, samples were merged again and scaled to perform a PCA and a new integration30. To identify and annotate the different clusters, we ran FindAllMarkers(). Clusters identified as doublets were removed. GSEA32, KEGG33 and Reactome34 were used for functional enrichment analysis with the package clusterProfiler31.

Cell-cell interactions and spot deconvolution
The CellChat database and R package36 with default parameters were used to separately identify biological interactions between annotated clusters in each condition. We focused on the relationships of TFCs and CT regions with the rest of areas by sources.use and target.use arguments.
Spots deconvolution of the three HT samples was performed using cell2location37 implemented in Python3. We used the annotation of the major cell types derived from our analysis of the sc-RNASeq HT published data27 (Supplementary Figure 3). As arguments, we specified 30 cells per location, a detection_alpha of 200, and 20000 epochs when mapping the cell signatures. Downstream plots depict the q05_cell_abundance_w_sf. Code is available at GitHub: https://github.com/endonutriHUPR/AITD_SpatialTranscriptomics.git.

For manuscripts utilizing custom algorithms or software that are central to the research but not yet described in published literature, software must be made available to editors and reviewers. We strongly encourage code deposition in a community repository (e.g. GitHub). See the Nature Portfolio guidelines for submitting code & software for further information.

Data

Policy information about availability of data

All manuscripts must include a data availability statement. This statement should provide the following information, where applicable:

- Accession codes, unique identifiers, or web links for publicly available datasets
- A description of any restrictions on data availability
- For clinical datasets or third party data, please ensure that the statement adheres to our policy

Spatial transcriptomics sequencing dataset generated in this study have been deposited in the Gene Expression Omnibus (GEO). Database with accession number GSE248205.

Research involving human participants, their data, or biological material

Policy information about studies with human participants or human data. See also policy information about sex, gender (identity/presentation), and sexual orientation and race, ethnicity and racism.

Reporting on sex and gender	Sex is specified in the Clinical data in Supplementary Table 1.
Reporting on race, ethnicity, or other socially relevant groupings	This is not relevant for our study
Population characteristics	This is not relevant for our study
Recruitment	From Hospital de La Princesa patients, we used thyroid tissue samples from AITD lesions and histologically normal parenchyma adjacent to other thyroid lesions from patients who underwent thyroidectomy at the Surgical Department of the Hospital Universitario la Princesa (registry number C0003922). All thyroid tissues were reviewed by an experienced pathologist and diagnosis was confirmed by an endocrinologist with commonly accepted clinical, laboratory, and histological criteria. Serum free thyroxine 4 (FT4), TSH, and levels of antibodies against thyroglobulin (TG), thyroperoxidase (TPO) and thyrotropin receptor (TSH-R) were measured.
Ethics oversight	This study was reviewed and approved by the Internal Ethics Review Committee of the Hospital de la Princesa (Committee

Register Number: 2796) and informed consent was obtained from all participants in accordance with the Declaration of Helsinki.

Note that full information on the approval of the study protocol must also be provided in the manuscript.

Field-specific reporting

Please select the one below that is the best fit for your research. If you are not sure, read the appropriate sections before making your selection.

[x] Life sciences [ ] Behavioural & social sciences [ ] Ecological, evolutionary & environmental sciences

For a reference copy of the document with all sections, see nature.com/documents/nr-reporting-summary-flat.pdf

Life sciences study design

All studies must disclose on these points even when the disclosure is negative.

Sample size	For spatial transcriptomics we were limited by the 10x Genomics slide (4 samples per slide). Due to this fact, we considered including 3 Hashimoto's thyroiditis, 3 Graves' disease and 2 controls (8 samples to spatial transcriptomics experiment). Tissue microarrays (TMA) were constructed with a total of 75 thyroid tissues (28 HT, 24 GD and 23 controls)
Data exclusions	We excluded those patients with other relevant pathologies.
Replication	To verify our main findings in the spatial transcriptomics data, we used 1) publicly available single cell data and 2) immunostaining techniques.
Randomization	Group patients were randomly selected
Blinding	Histology-based classification and immunostaining classification were done blindly by two independent experts.

Reporting for specific materials, systems and methods

We require information from authors about some types of materials, experimental systems and methods used in many studies. Here, indicate whether each material, system or method listed is relevant to your study. If you are not sure if a list item applies to your research, read the appropriate section before selecting a response.

Materials & experimental systems

n/a	Involved in the study	
[ ]	[x]	Antibodies
[ ]	[ ]	Eukaryotic cell lines
[ ]	[ ]	Palaeontology and archaeology
[ ]	[ ]	Animals and other organisms
[ ]	[x]	Clinical data
[ ]	[ ]	Dual use research of concern
[ ]	[ ]	Plants

Methods

n/a	Involved in the study	
[ ]	[ ]	ChIP-seq
[ ]	[ ]	Flow cytometry
[ ]	[ ]	MRI-based neuroimaging

Antibodies

Antibodies used	It is summarized in Supplementary Table 2
Validation	Antibodies were first validated at the recommended dilutions in testing tissue slices reported by the specific companies in order to confirm that each antibody were functional. Secondly, we validated the antibodies in our samples (healthy control and AITD samples). Finally, we increased the number of samples in our analysis using tissue microarrays, aiming to quantify each marker expression when applied.

Eukaryotic cell lines

Policy information about cell lines and Sex and Gender in Research

Cell line source(s)	We did not include cell lines in our analysis
Authentication	*Describe the authentication procedures for each cell line used OR declare that none of the cell lines used were authenticated.*
Mycoplasma contamination	*Confirm that all cell lines tested negative for mycoplasma contamination OR describe the results of the testing for mycoplasma contamination OR declare that the cell lines were not tested for mycoplasma contamination.*

Commonly misidentified lines (See ICLAC register)	*Name any commonly misidentified cell lines used in the study and provide a rationale for their use.*

Palaeontology and Archaeology

Specimen provenance	It is not relevant in our study
Specimen deposition	*Indicate where the specimens have been deposited to permit free access by other researchers.*
Dating methods	*If new dates are provided, describe how they were obtained (e.g. collection, storage, sample pretreatment and measurement), where they were obtained (i.e. lab name), the calibration program and the protocol for quality assurance OR state that no new dates are provided.*

☐ Tick this box to confirm that the raw and calibrated dates are available in the paper or in Supplementary Information.

Ethics oversight	*Identify the organization(s) that approved or provided guidance on the study protocol, OR state that no ethical approval or guidance was required and explain why not.*

Note that full information on the approval of the study protocol must also be provided in the manuscript.

Animals and other research organisms

Policy information about studies involving animals; ARRIVE guidelines recommended for reporting animal research, and Sex and Gender in Research

Laboratory animals	We did not include animals in our analysis
Wild animals	*Provide details on animals observed in or captured in the field; report species and age where possible. Describe how animals were caught and transported and what happened to captive animals after the study (if killed, explain why and describe method; if released, say where and when) OR state that the study did not involve wild animals.*
Reporting on sex	*Indicate if findings apply to only one sex; describe whether sex was considered in study design, methods used for assigning sex. Provide data disaggregated for sex where this information has been collected in the source data as appropriate; provide overall numbers in this Reporting Summary. Please state if this information has not been collected. Report sex-based analyses where performed, justify reasons for lack of sex-based analysis.*
Field-collected samples	*For laboratory work with field-collected samples, describe all relevant parameters such as housing, maintenance, temperature, photoperiod and end-of-experiment protocol OR state that the study did not involve samples collected from the field.*
Ethics oversight	*Identify the organization(s) that approved or provided guidance on the study protocol, OR state that no ethical approval or guidance was required and explain why not.*

Note that full information on the approval of the study protocol must also be provided in the manuscript.

Clinical data

Policy information about clinical studies

All manuscripts should comply with the ICMJEguidelines for publication of clinical research and a completedCONSORT checklist must be included with all submissions.

Clinical trial registration	It is not relevant for our study
Study protocol	*Note where the full trial protocol can be accessed OR if not available, explain why.*
Data collection	*Describe the settings and locales of data collection, noting the time periods of recruitment and data collection.*
Outcomes	*Describe how you pre-defined primary and secondary outcome measures and how you assessed these measures.*

Dual use research of concern

Policy information about dual use research of concern

Hazards

Could the accidental, deliberate or reckless misuse of agents or technologies generated in the work, or the application of information presented in the manuscript, pose a threat to:

No	Yes	
☒	☐	Public health
☒	☐	National security
☒	☐	Crops and/or livestock
☒	☐	Ecosystems
☒	☐	Any other significant area

Experiments of concern

Does the work involve any of these experiments of concern:

No	Yes	
☒	☐	Demonstrate how to render a vaccine ineffective
☒	☐	Confer resistance to therapeutically useful antibiotics or antiviral agents
☒	☐	Enhance the virulence of a pathogen or render a nonpathogen virulent
☒	☐	Increase transmissibility of a pathogen
☒	☐	Alter the host range of a pathogen
☒	☐	Enable evasion of diagnostic/detection modalities
☒	☐	Enable the weaponization of a biological agent or toxin
☒	☐	Any other potentially harmful combination of experiments and agents

Plants

Seed stocks	We did not include plants in our analysis
Novel plant genotypes	*Describe the methods by which all novel plant genotypes were produced. This includes those generated by transgenic approaches, gene editing, chemical/radiation-based mutagenesis and hybridization. For transgenic lines, describe the transformation method, the number of independent lines analyzed and the generation upon which experiments were performed. For gene-edited lines, describe the editor used, the endogenous sequence targeted for editing, the targeting guide RNA sequence (if applicable) and how the editor was applied.*
Authentication	*Describe any authentication procedures for each seed stock used or novel genotype generated. Describe any experiments used to assess the effect of a mutation and, where applicable, how potential secondary effects (e.g. second site T-DNA insertions, mosiacism, off-target gene editing) were examined.*

ChIP-seq

Data deposition

☐ Confirm that both raw and final processed data have been deposited in a public database such as GEO.

☐ Confirm that you have deposited or provided access to graph files (e.g. BED files) for the called peaks.

Data access links<br>*May remain private before publication.*	*For "Initial submission" or "Revised version" documents, provide reviewer access links. For your "Final submission" document, provide a link to the deposited data.*
Files in database submission	We did not include ChIP-seq experiments in our analysis
Genome browser session<br>(e.g. UCSC)	*Provide a link to an anonymized genome browser session for "Initial submission" and "Revised version" documents only, to enable peer review. Write "no longer applicable" for "Final submission" documents.*

Methodology

Replicates	*Describe the experimental replicates, specifying number, type and replicate agreement.*
Sequencing depth	*Describe the sequencing depth for each experiment, providing the total number of reads, uniquely mapped reads, length of reads and whether they were paired- or single-end.*
Antibodies	*Describe the antibodies used for the ChIP-seq experiments; as applicable, provide supplier name, catalog number, clone name, and lot number.*
Peak calling parameters	*Specify the command line program and parameters used for read mapping and peak calling, including the ChIP, control and index files used.*

Data quality	*Describe the methods used to ensure data quality in full detail, including how many peaks are at FDR 5% and above 5-fold enrichment.*
Software	*Describe the software used to collect and analyze the ChIP-seq data. For custom code that has been deposited into a community repository, provide accession details.*

Flow Cytometry

Plots

Confirm that:

- [ ] The axis labels state the marker and fluorochrome used (e.g. CD4-FITC).
- [ ] The axis scales are clearly visible. Include numbers along axes only for bottom left plot of group (a 'group' is an analysis of identical markers).
- [ ] All plots are contour plots with outliers or pseudocolor plots.
- [ ] A numerical value for number of cells or percentage (with statistics) is provided.

Methodology

Sample preparation	We did not include flow citommetry experiments in our analysis
Instrument	*Identify the instrument used for data collection, specifying make and model number.*
Software	*Describe the software used to collect and analyze the flow cytometry data. For custom code that has been deposited into a community repository, provide accession details.*
Cell population abundance	*Describe the abundance of the relevant cell populations within post-sort fractions, providing details on the purity of the samples and how it was determined.*
Gating strategy	*Describe the gating strategy used for all relevant experiments, specifying the preliminary FSC/SSC gates of the starting cell population, indicating where boundaries between "positive" and "negative" staining cell populations are defined.*

- [ ] Tick this box to confirm that a figure exemplifying the gating strategy is provided in the Supplementary Information.

Magnetic resonance imaging

Experimental design

Design type	We did not include MRI experiments in our analysis
Design specifications	*Specify the number of blocks, trials or experimental units per session and/or subject, and specify the length of each trial or block (if trials are blocked) and interval between trials.*
Behavioral performance measures	*State number and/or type of variables recorded (e.g. correct button press, response time) and what statistics were used to establish that the subjects were performing the task as expected (e.g. mean, range, and/or standard deviation across subjects).*

Acquisition

Imaging type(s)	*Specify: functional, structural, diffusion, perfusion.*
Field strength	*Specify in Tesla*
Sequence & imaging parameters	*Specify the pulse sequence type (gradient echo, spin echo, etc.), imaging type (EPI, spiral, etc.), field of view, matrix size, slice thickness, orientation and TE/TR/flip angle.*
Area of acquisition	*State whether a whole brain scan was used OR define the area of acquisition, describing how the region was determined.*

Diffusion MRI ☐ Used ☐ Not used

Preprocessing

Preprocessing software	*Provide detail on software version and revision number and on specific parameters (model/functions, brain extraction, segmentation, smoothing kernel size, etc.).*
Normalization	*If data were normalized/standardized, describe the approach(es): specify linear or non-linear and define image types used for transformation OR indicate that data were not normalized and explain rationale for lack of normalization.*

Normalization template

Describe the template used for normalization/transformation, specifying subject space or group standardized space (e.g. original Talairach, MNI305, ICBM152) OR indicate that the data were not normalized.

Noise and artifact removal

Describe your procedure(s) for artifact and structured noise removal, specifying motion parameters, tissue signals and physiological signals (heart rate, respiration).

Volume censoring

Define your software and/or method and criteria for volume censoring, and state the extent of such censoring.

Statistical modeling & inference

Model type and settings

Specify type (mass univariate, multivariate, RSA, predictive, etc.) and describe essential details of the model at the first and second levels (e.g. fixed, random or mixed effects; drift or auto-correlation).

Effect(s) tested

Define precise effect in terms of the task or stimulus conditions instead of psychological concepts and indicate whether ANOVA or factorial designs were used.

Specify type of analysis: ☐ Whole brain ☐ ROI-based ☐ Both

Statistic type for inference

(See Eklund et al. 2016)

Specify voxel-wise or cluster-wise and report all relevant parameters for cluster-wise methods.

Correction

Describe the type of correction and how it is obtained for multiple comparisons (e.g. FWE, FDR, permutation or Monte Carlo).

Models & analysis

n/a	Involved in the study
☐	☐ Functional and/or effective connectivity
☐	☐ Graph analysis
☐	☐ Multivariate modeling or predictive analysis

Functional and/or effective connectivity

Report the measures of dependence used and the model details (e.g. Pearson correlation, partial correlation, mutual information).

Graph analysis

Report the dependent variable and connectivity measure, specifying weighted graph or binarized graph, subject- or group-level, and the global and/or node summaries used (e.g. clustering coefficient, efficiency, etc.).

Multivariate modeling and predictive analysis

Specify independent variables, features extraction and dimension reduction, model, training and evaluation metrics.
